# First insight about the ability of specific glycerophospholipids to discriminate non-small cell lung cancer subtypes

**DOI:** 10.3389/fmolb.2024.1379631

**Published:** 2024-04-25

**Authors:** Julia Sieminska, Katarzyna Miniewska, Robert Mroz, Ewa Sierko, Wojciech Naumnik, Joanna Kisluk, Anna Michalska-Falkowska, Joanna Reszec, Miroslaw Kozlowski, Lukasz Nowicki, Marcin Moniuszko, Adam Kretowski, Jacek Niklinski, Michal Ciborowski, Joanna Godzien

**Affiliations:** ^1^ Metabolomics Laboratory, Clinical Research Centre, Medical University of Bialystok, Bialystok, Poland; ^2^ 2nd Department of Lung Diseases and Tuberculosis, Medical University of Bialystok, Bialystok, Poland; ^3^ Department of Oncology, Medical University of Bialystok, Bialystok, Poland; ^4^ 1st Department of Lung Diseases and Tuberculosis, Medical University of Bialystok, Bialystok, Poland; ^5^ Department of Clinical Molecular Biology, Medical University of Bialystok, Bialystok, Poland; ^6^ Department of Medical Patomorphology, Medical University of Bialystok, Bialystok, Poland; ^7^ Department of Thoracic Surgery, Medical University of Bialystok, Bialystok, Poland; ^8^ Altium International Sp. z o. o., Warszawa, Poland; ^9^ Department of Allergology and Internal Medicine, Medical University of Bialystok, Bialystok, Poland; ^10^ Department of Regenerative Medicine and Immune Regulation, Medical University of Bialystok, Bialystok, Poland; ^11^ Department of Endocrinology, Diabetology and Internal Medicine, Medical University of Bialystok, Bialystok, Poland

**Keywords:** non-small cell lung cancer NSCLC, adenocarcioma ADC, squamous call carcinoma SCC, oxidised glycerophosphatidylcholine oxPC, monoacylglycerophosphatidic acid LPA, lipidomics, metabolomics

## Abstract

**Introduction:** Discrimination between adenocarcinoma (ADC) and squamous cell carcinoma (SCC) subtypes in non-small cell lung cancer (NSCLC) patients is a significant challenge in oncology. Lipidomics analysis provides a promising approach for this differentiation.

**Methods:** In an accompanying paper, we explored oxPCs levels in a cohort of 200 NSCLC patients. In this research, we utilized liquid chromatography coupled with mass spectrometry (LC-MS) to analyze the lipidomics profile of matching tissue and plasma samples from 25 NSCLC patients, comprising 11 ADC and 14 SCC cases. This study builds upon our previous findings, which highlighted the elevation of oxidised phosphatidylcholines (oxPCs) in NSCLC patients.

**Results:** We identified eight lipid biomarkers that effectively differentiate between ADC and SCC subtypes using an untargeted approach. Notably, we observed a significant increase in plasma LPA 20:4, LPA 18:1, and LPA 18:2 levels in the ADC group compared to the SCC group. Conversely, tumour PC 16:0/18:2, PC 16:0/4:0; CHO, and plasma PC 16:0/18:2; OH, PC 18:0/20:4; OH, PC 16:0/20:4; OOH levels were significantly higher in the ADC group.

**Discussion:** Our study is the first to report that plasma LPA levels can distinguish between ADC and SCC patients in NSCLC, suggesting a potential role for LPAs in NSCLC subtyping. This finding warrants further investigation into the mechanisms underlying these differences and their clinical implications.

## 1 Introduction

Lung cancer is the most lethal cancer worldwide, causing 1.80 million deaths in 2020 (Bray et al., 2018). Two types of lung cancer are distinguished: non-small cell lung cancer (NSCLC) (85% cases) and small cell lung cancer. The most common histological subtypes of NSCLC are adenocarcinoma (ADC), squamous cell carcinoma (SCC), and large cell carcinoma (LCC). Within the NSCLC subtypes, ADC and SCC are predominant (Wang et al., 2020). ADC represents around 40% of NSCLC cases and is the most frequent subtype of lung cancer in non-smokers (Subramanian and Govindan, 2007; Kenfield et al., 2008; Schabath and Cote, 2019). In this subtype, cancer progression starts from lung glandular cells that produce mucin and surfactants. On the other hand, the SCC subtype, representing around 25% of NSCLC cases, is closely related to smoking and usually originates from the central areas of the lung bronchi (Campbell et al., 2016). Despite sharing several characteristics, they differ in clinical parameters and histopathology. The importance of the precise determination of the NSCLC subtype arises from the available treatment and its potential outcomes, which in turn are related to the presence of particular mutations prevalent in the majority of ADC and are rarely detected in SCC patients (Chen et al., 2019). The presence of mutation facilitates the selection of dedicated treatment, however, only around 60% of ADC and 50%–80% of SCC subjects have known oncogenic driver mutation (Yuan et al., 2019). The presence of these abnormalities allowed to propose several molecular targets for therapy, including vascular endothelial growth factor (VEGF), platelet-derived growth factor (PDGF), epidermal growth factor (EGF), insulin-like growth factor I (IGF-I) or anaplastic lymphoma kinase (ALK) (Ray et al., 2010). However, specific mutations were highly associated with ADC subjects. Moreover, for some therapies, even 60% of patients develop drug resistance (Yuan et al., 2019). As a result, despite availability of new treatments and strategies, still for many NSCLC patients’ classic histopathology-based therapies are the choice (Niemira et al., 2019), thus proper NSCLC subtyping is crucial (Ettinger et al., 2017).

Even though there are several findings describing differences between ADC and SCC, non-invasive early diagnostic techniques discriminating ADC and SCC are still not available. Recent lipidomics and metabolomics findings revealed a significant variation in lipids in NSCLC samples (Marien et al., 2015; Chen et al., 2018; Zhang et al., 2019; Fan et al., 2020; You et al., 2020; Jianyong et al., 2021; Kowalczyk et al., 2021).

Lipidomics emerged from metabolomics and can be defined as “the large-scale study of lipid species and their related networks and metabolic pathways that exist in cells or any other biologic system” (Sethi and Brietzke, 2017). Thanks to the untargeted approach, a very wide range of lipids can be analysed also in metabolomics studies. The most frequently used separation technique employed for determining lipid profiles is liquid chromatography (LC) coupled with accurate mass spectrometry (MS) (Fan et al., 2020; You et al., 2020).

In the work of our group, LC-MS-based untargeted metabolomics was implemented to discriminate NSCLC subtypes at different stages of the disease. This work covered patients with chronic obstructive pulmonary disease (COPD) as controls as well as ADC, SCC and LCC subjects in early and late stages. All analyses were performed on plasma and tissue samples, covering tumour and adjacent non-malignant lung tissue employing RP- and HILIC-LC/MS. It is important to highlight that this was not a lipidomics but a metabolomics study; however despite amino acids, most of the identified metabolites were lipids, including fatty acids, carnitines, lyso-glycerophospholipids (LPCs), glycerophospholipids, plasmalogens, sphingomyelins (SMs), and glycerophospho (N-acyl)ethanolamines (Kowalczyk et al., 2021). Fan et al. performed RP-LC/MS analyses over the lung cancer tissue and benign lung tissue, both paired with distal noncancerous tissue from the same patient. They reported and described changes in the lipid profile of lung cancer but also provided receiver operating characteristic (ROC) curve analysis of combinational lipid markers to assist in the disease diagnosis (Fan et al., 2020). A similar design was used by You et al., who also analysed the lung cancer tissue and benign lung tissue and paired distal and adjunct noncancerous tissue. They employed RP-LC/MS to explore metabolic reprogramming of lung cancer and to distinguish NSCLC subtypes. They found changes among different metabolite classes associated with the alterations in energy and purine metabolism, biosynthesis of amino acids, membrane lipid metabolism, and glutamine and cysteine and methionine metabolism (You et al., 2020). Zhang et al. used MS imaging to discriminate between post-operative NSCLC tumours and paired normal tissues. This research covered also the recognition of mutations of epidermal growth factor receptor (EGFR), which is crucial from a diagnostic and therapeutic perspective. Glycerophospholipids were found to differentiate between ADC and SCC subtypes, but also between EGFR-mutated-positive and EGFR-wild-type tissue (Zhang et al., 2019). Marien et al. used direct infusion and 2D-imaging MS to profile glycerophospholipids in malignant and non-malignant lung tissue of NSCLC patients. Their results revealed decreased levels of sphingomyelins and glycerophosphoserines (PSs) and elevated levels of glycerophosphoinositols (PIs), glycerophosphoethanolamines (PEs) and glycerophosphocholines (PCs) (Marien et al., 2015).

All these publications pointed to the alterations in the lipid profile, including glycerophospholipids. Glycerophospholipids are susceptible to oxidation, and oxidative stress was reported as one of the underlying processes accompanying NSCLC; therefore, in our previous study, we decided to explore the profile of oxidised glycerophosphocholines (oxPCs) in lung cancer patients (Godzien et al., 2024). We proved, that these early oxidation products are altered in the NSCLC patients in comparison to COPD controls. In this companion paper, we decided to explore oxPCs changes deeper, making direct comparison between NSCLC subtypes but also combining information about oxPCs with lipid profiles of SCC and ADC patients. Considering the high interconnectivity between distinct lipid classes along with the fact that oxidised lipids originate from the native, non-oxidised lipids, we believed that such a strategy can benefit in data interpretation.

OxPCs are currently deeply investigated since their role went beyond oxidation by-products, and nowadays, they are recognised as important molecules with multiple functions. They were associated with cardiovascular diseases (Paynter et al., 2018), diabetes (Godzien et al., 2019), neurogenerative disorders (Okuzumi et al., 2019) and cancer (López-López et al., 2020). Oxidation of PCs can lead to the formation of different types of epi-lipids, a subset of the natural lipidome formed by either enzymatic or non-enzymatic modifications such as, e.g., oxidation, nitration, or sulfation with their own biological functions. oxPCs cover mildly oxidised long-chain oxPCs (LCh-oxPCs), truncated short-chain oxPCs (SCh-oxPCs), and cyclised oxPCs. These molecules have different structures and, therefore, different bio-activities: each group of oxPCs can have a distinct function, or they might have contradictory effects. Moreover, pleiotropic functions of oxPCs were also observed. Because of this, exploration of the involvement of oxPCs in different pathologies is so important.

As pointed above, NSCLC is the most lethal, and, therefore, one of the most frequently studied cancer; however, despite all available knowledge, new diagnostic tools are needed. To propose valid and reliable methods for unequivocal subtypes discrimination, we need to explore and understand metabolic alterations underlying the ADC and SCC. In this study, we have focused on identification of lipids, including oxPCs, in plasma and tissue samples that would allow discrimination between the SCC and ADC subtypes.

## 2 Materials and methods

### 2.1 Chemical and reagents

Ultrapure water was used to prepare all the aqueous solutions and was obtained “in-house” from a Milli-Q Integral three system (Millipore, SAS, Molsheim, France). Zomepirac sodium salt, formic acid, LC-MS-grade methanol and acetonitrile, and LC-grade ethanol were purchased from Sigma-Aldrich Chemie GmbH (Steinheim, Germany).

### 2.2 Cohort study

Samples were obtained from patients undergoing surgical treatment for primary NSCLC at the Thoracic Surgery Department of Medical University of Bialystok Clinical Hospital in Poland. The study was approved by the Ethics Committee of the Medical University of Bialystok (R-I-003/262/2004, R-I-002/296/2018 and APK 002 5 2021) and performed in accordance with the Declaration of Helsinki. Before collecting the samples, written informed consent for specimen collection was obtained from all participants.

In this project we included 122 tissue samples collected from 61 NSCLC patients. There were two tissue samples per patient: tumour tissue and adjacent non-malignant control tissue. Among these 61 patients, 25 were classified as ADC and 36 as SCC subjects. Plasma analyses covered samples collected from 101 NSCLC patients, among which 41 were diagnosed with ADC and 60 with SCC. Analyses of clinical records revealed that 25 patients were common between the plasma and tissue cohorts, covering 11 ADC and 14 SCC subjects. In this study, we focused on these 25 subjects, performing joined analyses of plasma and tissue lipids. Basic clinical parameters describing the available cohort and selected sub-cohort are summarised in Table 1.

**TABLE 1:** Basic clinical parameters characterising three cohorts of patients enrolled in the study.

Plasma metabolic fingerprinting whole cohort/subcohort			
Patients’ characteristic	NSCLC	ADC	SCC
Age [years] (median)	63.0/62.0	62.0/62.0	63.5/61.0
Q1	58.0/56.0	58.0/55.5	58.8/56.5
Q3	69.0/66.0	68.0/67.5	69.0/65.8
BMI (median)	25.47/24.69	25.99/25.00	25.4/24.07
Q1	23.62/23.50	24.17/23.9	23.4/23.3
Q3	27.76/26.00	28.09/25.7	27.23/25.9
Gender [F/M]	[29/72]/[5/20]	[15/26]/[3/8]	[14/46]/[2/12]
pTNM			
IIA	24/6	8/4	16/2
IIB	33/13	9/5	24/8
IIIA	18/6	8/2	10/4

Tumour tissue metabolic fingerprinting whole cohort/subcohort			
Patients’ characteristic	NSCLC	ADC	SCC
Age [years] (median)	63.0/63.0	62.0/62.0	63.5/63.5
Q1	56.0/56.0	55.0/55.5	57.5/59.3
Q3	69.0/69.0	69.0/67.5	69.0/68.3
BMI (median)	24.93/25.00	25.00/25.00	24.53/25.06
Q1	23.41/23.83	23.53/23.90	23.39/23.91
Q3	26.09/25.69	26.37/25.73	25.98/25.60
Gender [F/M]	[13/48]/[7/18]	[6/19]/[3/8]	[7/29]/[4/10]
pTNM			
IIA	21/5	9/3	12/2
IIB	20/10	8/5	12/5
IIIA	20/10	8/3	12/7

pTNM, pathological tumour-nodemetastasis. For pTNM staging number of patients assigned to given stage is provided for the whole cohort and subcohort.

Whole blood was collected in 9 mL vacuum system tubes with K_2_EDTA as an anticoagulant. After gentle mixing, plasma was separated by centrifugation at 1300 *g* for 20 min at room temperature. Plasma fractions (0.5 mL each) were then collected in Eppendorf tubes and stored at −80°C until analysis.

Collected tissue samples were histologically reviewed and classified. After lung tumour resection, whole specimen was examined macroscopically by the pathologist to determine the exact tumour localization, presence or absence of macroscopic residual tumour, presence or absence of macroscopic infiltration of pulmonary pleura, macroscopic evaluation of possible presence of necrosis in the tumour centre. The pathologist cut the exact tissue samples that represent the tumour centre and tumour margin. Moreover, the pathologist determined the possibility to collect adjacent pulmonary tissue (referred as normal tissue): if the distance from the tumour border was greater than 2 cm, the pathologist cut the samples of adjacent tissue. Then, immediately after resection, study nurses from the Biobank put the tissue samples alternately into cryotubes for vapor phase of liquid nitrogen (fresh frozen samples) and into tubes with 10% buffered formalin (formalin-fixed samples). The exact time of resection start, vessel ligation, resection end, and tissue sample preservation were recorded and documented. The details of tissue samples collection in the clinical setting–macroscopic evaluation resected specimens were described previously (Ciereszko et al., 2022), together with the biobanking conditions and Standard Operating Procedures (Niklinski et al., 2017; Michalska-Falkowska et al., 2023). Cancer stages were determined following pathological tumour-node-metastasis (pTNM) staging. All tissue samples were frozen and stored at −80°C until the day of analyses. Sample collection, quenching and storage were performed following approved biobanking standards (Niklinski et al., 2017).

### 2.3 Sample preparation

Plasma samples were prepared using the previously described method (Daniluk et al., 2019). On the day of analysis, samples were thawed on ice. For protein precipitation and metabolite extraction, one plasma sample volume was mixed with three volumes of ice-cold methanol/ethanol (1:1) containing 1 ppm of Zomepirac (internal standard IS). After extraction, samples were stored on ice for 10 min and centrifuged at 21,000 × *g* for 20 min at 4°C. The supernatant was filtered into a glass HPLC-vial through a 0.22 μm nylon filter (ThermoFisher Scientific, Waltham, Massachusetts, United States of America).

Tissue samples were prepared following the previously described method (Ciborowski et al., 2017). On the day of analysis, samples were thawed on ice. Ten milligrams of lung tissue sample were placed in an Eppendorf tube with two stainless steel beads (5 mm) and 200 μL of ice-cold 50% methanol. Samples were homogenised for 8 min at 30 Hz using Tissue Lyser (LT; Qiagen Hilden, Germany). After homogenisation, beads were removed and 200 μL of ice-cold acetonitrile containing 1 ppm of IS was added to the sample. Metabolites were extracted by vortex-mixing the samples for 1 hour. After extraction, samples were centrifuged at 21,000 × *g* for 20 min at 20°C. After centrifugation, the supernatant was filtered through a 0.22 μm nylon filter (ThermoFisher Scientific, Waltham, Massachusetts, United States of America). Extraction blank was prepared following the same procedure as biological samples, but without tissue, and was analysed together with biological samples.

Quality control (QCs) samples were prepared by mixing equal volumes of all raw plasma samples and all extracts for tissue samples. QCs were treated like the rest of the samples and injected at the beginning of the batch (10 injections) to equilibrate the system and every ten samples further to monitor the stability of the measurement (Godzien et al., 2014).

### 2.4 Analytical set-up

Plasma analyses were performed using a 6546 iFunnel ESI-Q-TOF (Agilent Technologies, Germany) coupled with a 1290 Infinity UHPLC system (Agilent Technologies, Germany) with a degasser, quaternary pump and thermostated autosampler.

Tissue analyses were performed using a 6545 iFunnel ESI-Q-TOF (Agilent Technologies, Germany) coupled to a 1290 Infinity UHPLC system (Agilent Technologies, Germany) with a degasser, binary pump and thermostated autosampler.

Plasma and tissue samples were analysed in both polarity modes. During all analyses, two reference compounds were used: *m/z* 121.0509 (protonated purine) and *m/z* 922.0098 (protonated hexakis (1H,1H,3H-tetrafluoropropoxy)phosphazine (HP-921)) for positive ionisation mode and *m/z* 112.9856 (proton abstracted trifluoroacetic acid anion) and *m/z* 966.0007 (formate adduct of HP-921) for negative ionisation mode. These masses were continuously infused into the system to allow internal constant mass correction during data acquisition.

### 2.5 Metabolic profiling

#### 2.5.1 Plasma analyses

Four microliters of each sample were injected into a thermostated at 60°C Zorbax Extend C18 column (RRHT 2.1 × 50 mm, 1.8 μm Agilent Technologies, Santa Clara, California, United States of America). The flow rate was 0.6 mL/min with solvent A (water with 0.1% formic acid) and solvent B (acetonitrile with 0.1% formic acid). The chromatographic gradient started at 50% of phase B, then increased the amount of phase B to 80% (from 1 to 6 min) and 100% (from 6 to 8 min). Finally, the system was re-equilibrated by reverting phase composition to initial conditions (50% phase B) in 0.5 min, which was kept from 8.5 to 10 min. The mass spectrometer was operated in full scan mode. Data were acquired at *m/z* ranging from 50 to 1,000 at the scan rate of 1.0 scans per second. Nebulizer pressure was set at 52 psig, nozzle voltage at 1,000 V, and capillary voltages at 3,000 and 4,000 V in the positive and negative ion mode, respectively.

All samples were analysed in scan mode in both polarity modes. Then, a subset of 70 samples was analysed in negative ion mode using iterative exclusion data-dependent analysis (IE-DDA). Precursor ions were fragmented using ramped collision energy, adjusted for each molecule according to its *m/z*. The first injection was performed as a conventional data-dependent MS/MS analysis, where the top three most abundant precursors were selected for fragmentation, considering active exclusion lists. In subsequent injection, precursors selected for MS/MS fragmentation in the previous injection were excluded on a rolling basis with 20 ppm mass error tolerance and 0.5 min retention time tolerance. Five iterative-MS/MS runs were set for each sample, resulting in 350 measurements.

#### 2.5.2 Lung tissue analyses

One μL of the extracted sample was injected into a thermostated at 60°C Zorbax Eclipse Plus C8 column (RRHD 2.1 × 150 mm, 1.8 μm Agilent Technologies, Santa Clara, California, United States of America). The flow rate was 0.6 mL/min with solvent A (water with 0.1% formic acid) and solvent B (acetonitrile with 0.1% formic acid). The gradient started at 25% phase B and increased to reach 95% of phase B in 14 min. This proportion was kept for 1 min, and after that, the gradient returned to starting conditions (25% phase B) in 0.1 min and was maintained for 4.9 min to re-equilibrate the system before the next injection. The mass spectrometer was operated in full scan mode from *m/z* 50 to 1,000. The capillary voltage was set to 3,000 V for ESI+, and 4,000 V for ESI- mode; the drying gas flow rate was 12 L/min, temperature 250°C and gas nebuliser 52 psig. Fragmentor voltage was 250 V for both ESI modes.

### 2.6 Determination of protein content in lung tissue

The precipitated proteins were suspended in radioimmunoprecipitation assay (RIPA) buffer and then denatured at 60°C and sonicated for 30 min in a water bath. The samples were then centrifuged for 15 min at 14,000 × *g*. Protein concentration was measured with the Pierce BCA Protein Assay Kit (Thermo Fisher Scientific) according to the included protocol.

### 2.7 Data processing

#### 2.7.1 Plasma metabolic profiling

Plasma data was reprocessed twice: searching for oxPCs and for other lipids. MS1 data were reprocessed using a targeted approach and searching solely for oxPCs (Godzien et al., 2019). Data from iterative exclusion data-dependent analyses were used to confirm the annotation of oxPCs. For this, we searched for known fragmentation patterns (Gil de la Fuente et al., 2018). Moreover, all annotations were confirmed using retention time (RT) to compare the elution order between different oxPCs and their non-oxidised precursors. The list of 45 oxPCs was defined, covering 13 LCh-oxPCs and 32 SCh-oxPCs (including iso-forms). RT and mass data pairs were used as input criteria to find oxPCs. Processing was performed using an algorithm “Find by Ion” in Mass Hunter Profinder software (Agilent, B.10.00). The integration of all extracted peaks was manually curated and corrected if necessary.

Data from IE-DDAs were reprocessed using the untargeted approach in Lipid Annotator software (Agilent, B.01.00). For lipid annotation, a fragmentation-based (MS/MS) library was used. The resulting data comprise the *m/z* of all the precursors identified as lipids, their corresponding RT, and their classification into lipid categories and classes. During the reprocessing, allowed ions covered [M-H]^−^, [M + HCOOH-H]^−^, and [M + Cl]^−^. The Q-Score was set at ≥ 50, the mass deviation was established as ≤ 20 ppm, the fragment score threshold was fixed as ≥ 30, and the total score was set at ≥ 60. The list of annotated lipids was then used in Mass Hunter Profinder software (Agilent, B.10.00) for the targeted search, where a sophisticated algorithm searched selected ions across MS1 data. Data were reprocessed considering ions [M + H]^+^, [M + Na]^+^, [M + K]^+^, [CHNaO_2_]^+^ and [C_2_H_2_Na_2_O_4_]^+^ in positive ionisation mode and [M-H]^-^, [M + HCOO]^-^, [M + Cl]^-^, [C_2_HF_3_O_2_], [C_3_H_2_F_3_NaO_4_] in negative ionisation mode. Neutral loss of [C_2_H_4_O_2_] and [CH_3_] was used in negative ion mode, while water loss was considered in both polarities. The maximum permitted charge state was double. Alignment was performed based on *m*/*z* and RT similarities within the samples. Parameters applied were 0.5% and 0.20 min for the RT window and 20 ppm and 2 mDa for mass tolerance. These were selected based on the assessment of raw data.

#### 2.7.2 Tissue metabolic profiling

Raw data was reprocessed, searching for oxPCs implementing the targeted approach (Godzien et al., 2019). The list of 45 oxPCs was defined, covering 13 LCh-oxPCs and 32 SCh-oxPCs (including iso-forms). Annotation of these oxPCs was done in previous projects based on the data-independent analysis (DIA), and incorporated into in-house built library. RT and mass data pairs of annotated oxPCs were used as input criteria to search them in MS1 data. Processing was performed using the same algorithm “Find by Ion” in Mass Hunter Profinder software (Agilent, B.10.00) as described above. The integration of all extracted peaks was manually curated and corrected if necessary.

### 2.8 Data analysis

The acquired data underwent evaluation through a Quality Assurance procedure. Lipids displaying a Relative Standard Deviation (RSD) of signals in QC samples below 30% were considered reliably measured and retained for subsequent analyses. An additional filter was applied to keep signals detected in at least 75% of samples in at least one sample group.

Before the statistical analyses, reprocessed data were normalised. Data from plasma analyses were normalised solely to the internal standard to minimise analytical drift. Data from tissue analyses were normalised to the internal standard and the protein content to minimise differences between different pieces of tissue.

Data analyses were done over each matrix, matching the patients, plasma, and tissue samples. Statistics were computed by comparing ADC and SCC subtypes. Differences between compared groups were described with *p*-value and percentage of change, where a positive value indicated an increase in ADC patients compared to the SCC patients, while a negative value illustrated a decrease of the signal in ADC patients compared to the SCC patients. Furthermore, we calculated the median, Q1 and Q3 for the level of each oxPC for each comparison (Table 2). Percentage of change, median, Q1 and Q3 were computed using Excel (Microsoft). *p*-value was computed employing a non-parametric Mann-Whitney test and then corrected by applying Benjamini–Hochberg FDR using in-house-built Matlab scripts (version 2020a, Mathworks, Natick, MA, United States of America). FDR was performed only for metabolites with the absolute percentage of change equal or greater than 25%. Random Forest analysis was performed using the in-house-written code in RStudio software (version 2023.12 + 369, PBC, Boston, MA, United States of America). Used Matblab scripts and R code are provided in the Supplementary Material (Data Sheet 2). The ROC curve analysis was used to test the discriminating metabolites as potential biomarkers and to evaluate their performance. Areas under the curve (AUCs) were calculated by implementing Random Forest in MetaboAnalyst (version 5.0).

**TABLE 2:** Lipids discriminate significantly between plasma and tumor tissue samples from ADC and SCC patients.

Compound	raw *p*-value (corrected *p*-value)	Gini importance score	% of change	ADC	SCC
				Median (Q1-Q3)	
tumour tissue					
PC 16:0/4:0; CHO	0.0331 (0.1214)	0.1088	−59.3	0 (0–546)	497 (399–609)
PC 16:0/18:2	0.0231 (0.1214)	0.1245	+40.5	184,585 (172,541–226,863)	113,284 (91,216–162,270)
plasma					
PC 16:0/20:4; OOH	0.0068 (0.0466)	0.0944	+25.1	182,738 (159,665–218,149)	136,157 (118,852–152,437)
PC 18:0/20:4; OH	0.0199 (0.0466)	0.0904	+74.7	260,157 (212,240–311,125)	188,303 (133,987–225,592)
PC 16:0/18:2; OH	0.0172 (0.0466)	0.1039	+95.4	244,459 (213,149–444,861)	157,436 (118,621–236,128)
LPA 18:1	0.0172 (0.0862)	0.0934	−59.3	15,060 (13,913–18,269)	23,460 (17,022–41,625)
LPA 18:2	0.0306 (0.0982)	0.0965	−37.5	15,713 (12,755–16,926)	23,896 (19,512–33,909)
LPA 20:4	0.0040 (0.0405)	0.1476	−88.7	15,689 (13,393–26,941)	87,239 (36,585–321,949)

+means an increase in ADC, group in comparison to SCC, group.

- means a decrease in ADC, group in comparison to SCC, group. % of change and median (Q1-Q3) are provided for the signal intensity for a given lipid.

### 2.9 Pathway analysis

Pathway analysis was performed based on the LIPID MAPS^®^ reaction explorer. Different lipid species were linked based on the reactions from various sources, including scientific literature, the lipid research community, and other existing databases such as Rhea, WikiPathways, KEGG, Ecocyc, and MetaCyc. In the analysis, we included all detected and annotated lipids.

## 3 Results and discussion

### 3.1 General concerns

Baseline characteristics of ADC and SCC patients is summarised in Table 1. Enrolled subjects were matched in basic anthropometric measurements, and no between-group statistical differences were observed. The only concern is with the lack of gender balance since more men than women were enrolled. However, this corresponds to a greater number of diagnosed men than women at the time of sample collection.

All samples were analysed in both polarity modes. Metabolite annotation was performed using information acquired in negative ion mode since this polarity provides more details, such as the exact composition of fatty acids. Statistical analysis was performed on the data acquired in positive ion mode because more abundant and, therefore, more reproducible signals for oxPCs were obtained in this polarity mode (Godzien et al., 2024).

Analyses performed for 122 tissue samples collected from 61 NSCLC patients, covering two samples per patient: adjacent non-cancerous lung tissue and lung tumour tissue, allowed measurement and annotation of 16 oxPCs: 15 SCh-oxPCs and only 1 LCh-oxPCs. Analyses performed for 101 plasma samples allowed measurement and annotation of 13 oxPCs: 5 SCh-oxPCs and 8 LCh-oxPCs. Automatic annotation based on IE-DDA provided information about 120 plasma lipids, covering: 17 sphingolipids (2 ceramides (Cer) and 15 SMs), 54 monoacylglycerophospholipids (5 LPAs, 30 LPCs, 14 LPEs, 4 LPIs and 1 LPGs), 31 diacylglycerophospholipids (17 PCs, 9 PEs, 4 PIs and 1 PSs), 11 ether-glycerophospholipids (4 ether PCs and seven ether PEs) and seven fatty acids. Although the maximum allowed error for annotation was set to 20 ppm, the greatest mass error was 1.3 Da, with the average error for all 294 annotated lipids of 0.36 Da. The obtained matrix contained 8% of missing values which were present only in 12 out of 165 features.

### 3.2 Differences in the lipid profile between ADC and SCC subjects

In recent years, lipidomics (especially the untargeted approach) has emerged as a promising tool for medicine, allowing the selection of potential biomarkers but also providing insight into the mechanisms underlying different diseases. For this reason, we decided to apply the LC-MS method to search for lipids differentiating main NSCLC subtypes.

We analysed tissue and plasma samples collected from patients diagnosed with ADC or SCC. Univariate Mann-Whitney analysis, followed by Benjamini–Hochberg post-correction, allowed the selection of eight discriminating variables, which are listed in Table 2. We focused on lipids, for which the raw *p*-value was below 0.05. However, considering that the corrected *p*-value was not significant for all of these lipids, we decided to support the statistical evaluation of pre-selected variables by Random Forest and ROC analysis. The given lipid was considered statistically significant if it met three conditions: the *p*-value was below 0.05 and ranked in the top 20 variables according to the Gini score (Table 2), and the AUC for ROC was above 0.75. Random Forest analysis performed for 2000 trees resulted in the model with an out-of-bag (OOB) error rate of 0.32. Among the top 20 most important variables were all eight significant lipids: four of them were in the top 10.

All statistically significant lipids belong to glycerophospholipids. As far as plasma samples are concerned, we found differences in the level of several oxPCs and LPAs. In non-malignant lung samples, there were no discriminating lipids, while in tumour tissue samples, the only lipids discriminating these two subtypes were PC 16:0/18:2 and PC 16:0/4:0; CHO.

**FIGURE 1:**
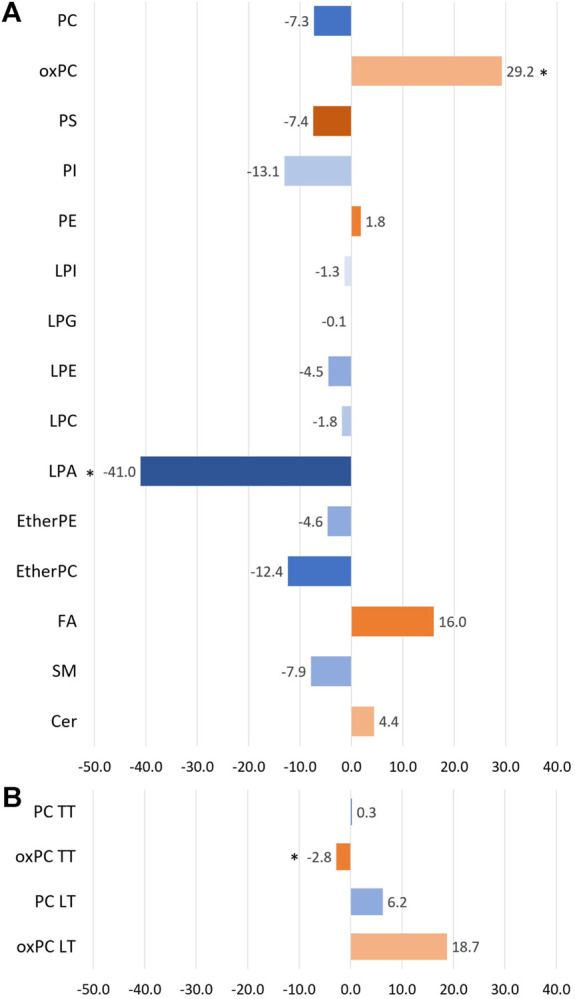
The bar plot illustrates the percentage of averaged change between the ADC and SCC patients across the different lipid classes. Panel **(A)** portraits the changes observed in plasma samples, while panel **(B)** shows the changes observed for tumour tissue samples (TT) and healthy lung tissue (LT). A negative value means a decrease in the ADC group compared to the SCC group (marked with bluish colours), and a positive value reflects an increase in the ADC group compared to the SCC group (marked with orangish colours). * indicates the groups of lipids for which observed changes were statistically significant.

Before discussing discriminating lipids, we want to comment on the lipid profile obtained for NSCLC patients. Averaged changes observed for each lipid class are graphically represented in Figure 1. Only three types of lipids, marked with the asterisk, were found to be statistically different between the two lung cancer subtypes. However, as can be noticed, some of the changes, despite their magnitude, are not significant. Not all lipids belonging to a particular class exhibited the same direction of change.

**FIGURE 2:**
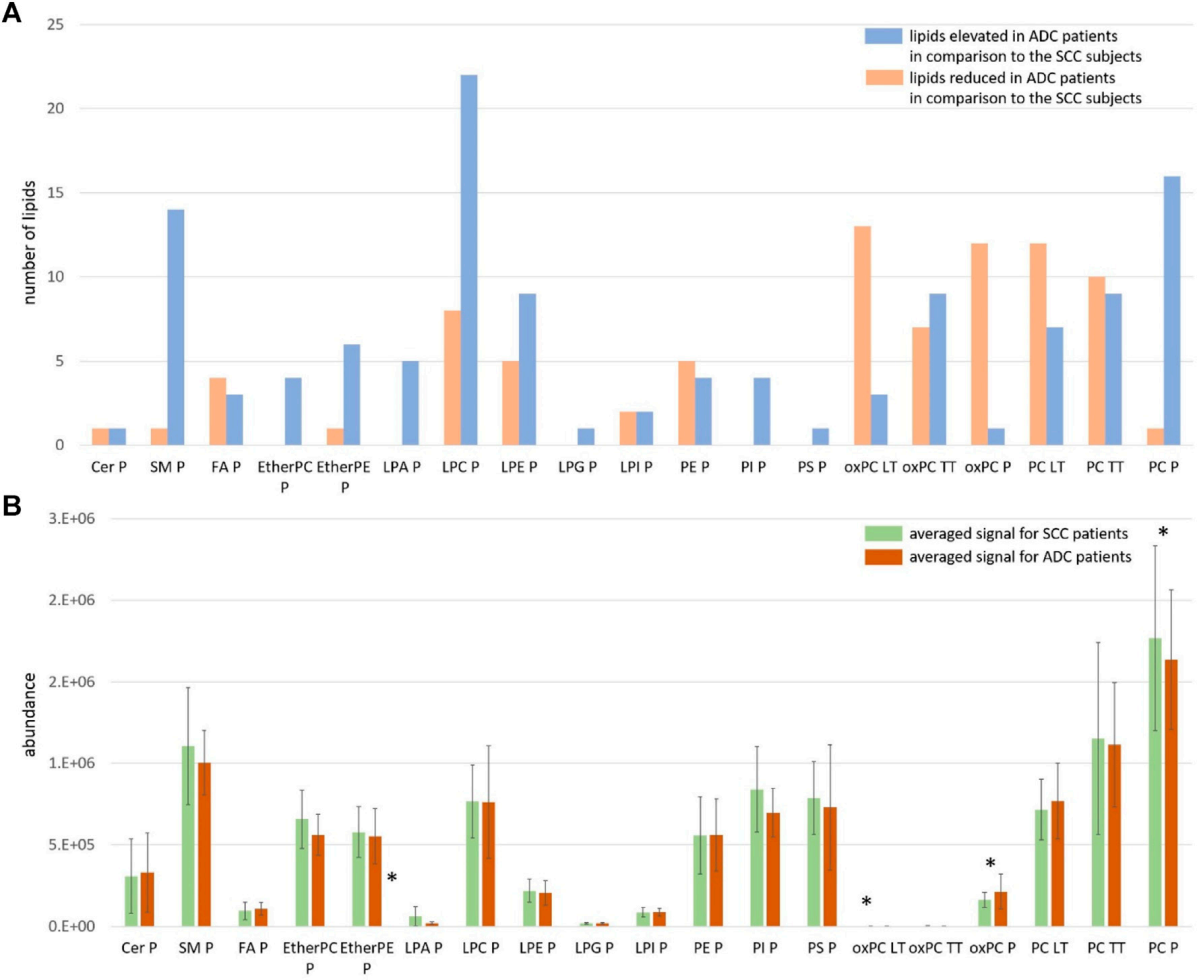
Panel **(A)** The number of measured and identified lipids for each class, assigned as elevated (orange colour) and reduced (blue colour) in ADC patients in comparison to the SCC subjects; Panel **(B)** The averaged signal for lipids belonging to each class measured for SCC patients (green colour) and ADC patients (brown colour). The y-axes in the graph represent the abundance of metabolites. The whiskers show the standard deviation of the averaged signal. * indicates the groups of lipids for which observed changes were statistically significant; LT: healthy lung tissue; TT: tumour tissue; P: plasma.

For this reason, we visualised the data and compared the number of individual lipid species in each class that was increasing and decreasing in the ADC compared to the SCC group. Results of this visualisation are presented in Panel A of Figure 2. The first observation leads to the conclusion that most noted changes correspond to the decrease of the signal in ADC patients. It is the case of SMs, plasma PCs, PI, PS, etherPCs and etherPEs, LPA and LPG. An increased signal was observed for oxPCs in healthy lung tissue; however, this change was insignificant for oxPCs in plasma samples. Very different changes, corresponding almost equally to both the increase and the decrease of the signal, were observed mainly for FAs, LPEs, LPIs, PEs, and tumour tissue PCs. Panel B of Figure 2 illustrates the average signal for lipids belonging to a particular class for ADC patients (brown colour) and SCC subjects (green colour). It is essential to highlight that this data can be used to compare ADC and SCC subjects within a given lipid group. A comparison of absolute values for lipids measured in tissue and plasma cannot be performed. This data corresponds to the measured signal and not the total quantitative value. Observed averaged changes are in concordance with the direction of change of lipids: SMs, plasma PCs, PI, PS, etherPCs and etherPEs, LPA and LPG are reduced in ADC patients in comparison to the SCC subjects, while in plasma oxPCs, PCs detected in healthy lung tissue and Cer the signal is higher in ADC patients.

**FIGURE 3:**
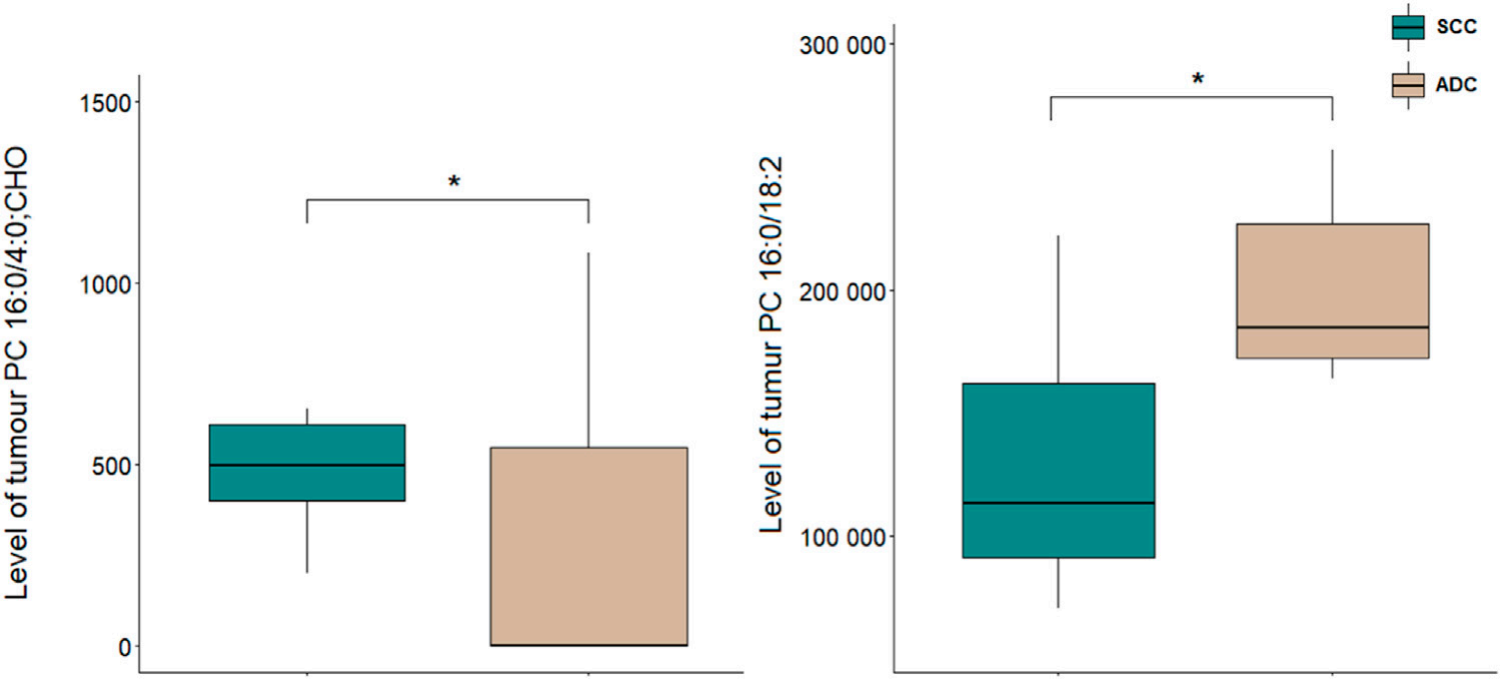
Levels of lipid discriminating between tumour tissue samples of ADC and SCC sam-ples. *p < 0.05. The *y*-axes in the graph represent the abundance of metabolites. The whiskers show the minimum and maximum values. The bottom and top of the box are the 25^th^ and 75^th^ percentiles, and the line inside the box is the 50^th^ percentile (median).

A comparison of NSCLC patients showed that PC 16:0/18:2 was increased in ADC group in comparison to SCC group, and PC 16:0/4:0; CHO was decreased in ADC group compared to SCC group (Figure 3).

**FIGURE 4:**
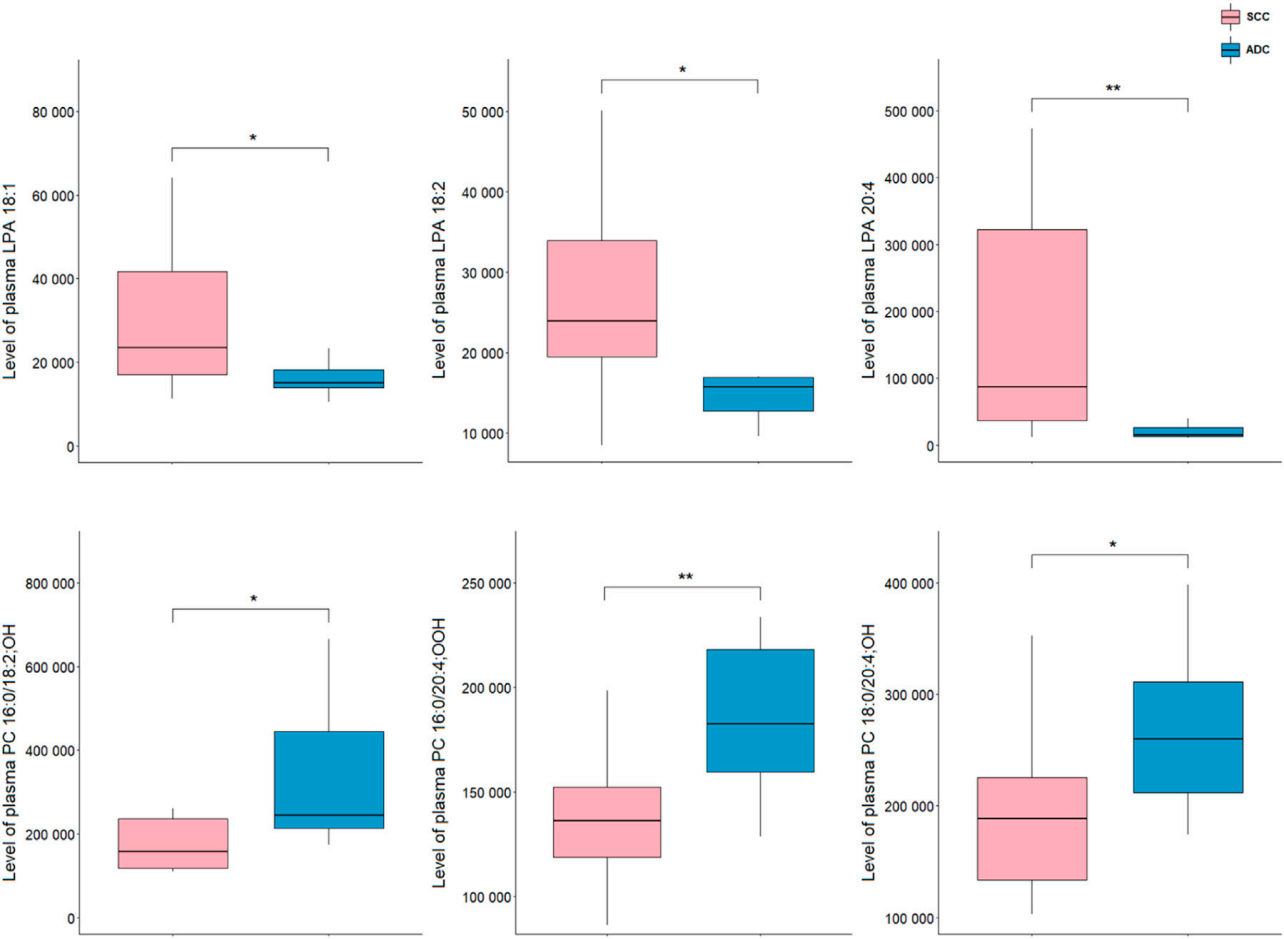
Levels of lipid discriminating between plasma samples of ADC and SCC samples. *p < 0.05; **p < 0.01. The *y*-axes in the graph represent the abundance of metabolites. The whiskers show to the min and max values. The bottom and top of the box are the 25^th^ and 75^th^ percentiles, and the line inside the box is the 50^th^ percentile (median).

Analysis of plasma samples showed a higher number of significant metabolites as compared to tissue results. All differentiating lipids belong to two classes of glycerophospholipids, namely, LPAs and oxPCs. A comparison of NSCLC patients showed that the levels of LPA 20:4, LPA 18:1, and LPA 18:2 in ADC group were significantly decreased compared with SCC, whereas the levels of PC 16:0/18:2; OH, PC 18:0/20:4; OH, PC 16:0/20:4; OOH were significantly increased (Figure 4).

**FIGURE 5:**
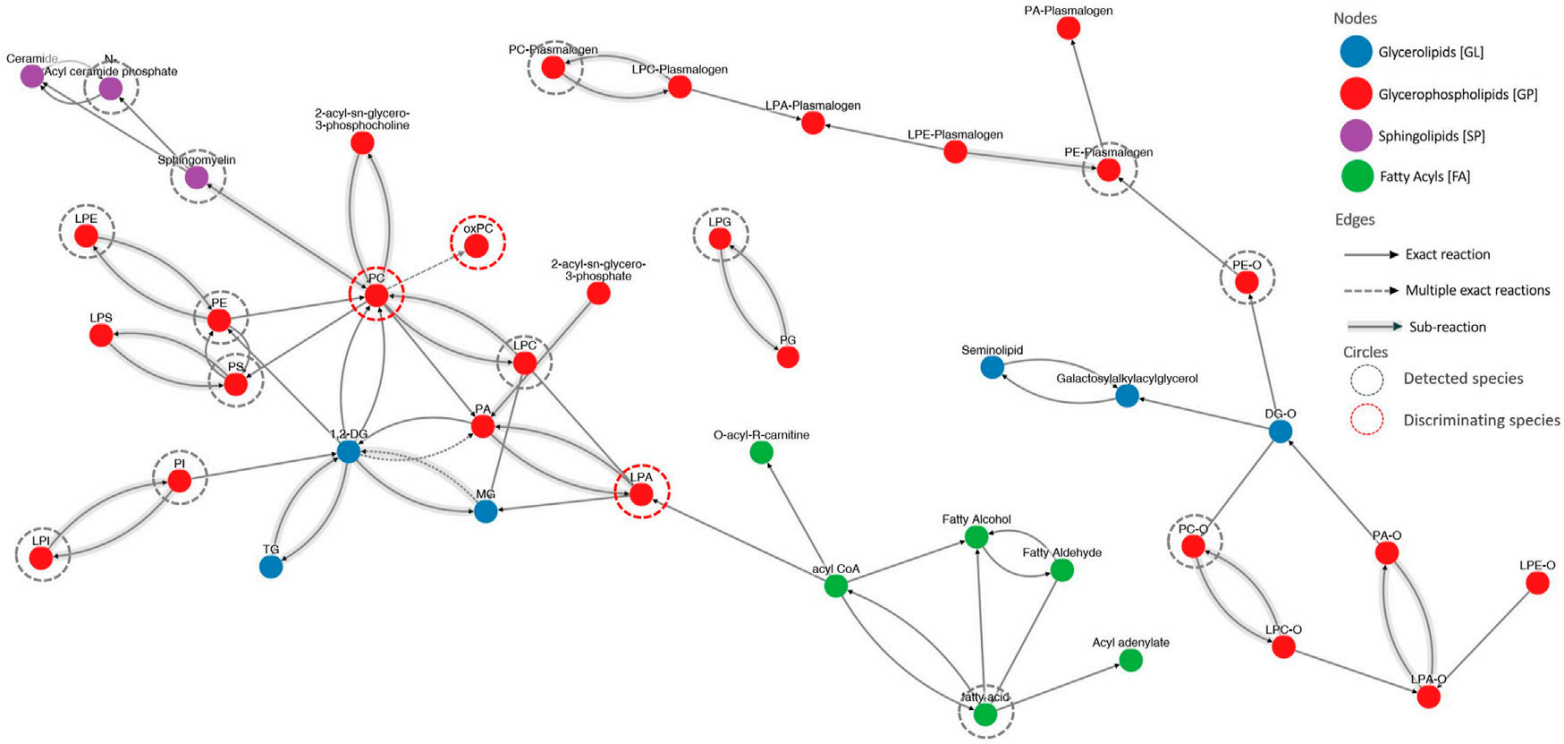
The visualization of lipid connectivity is based on the biochemical reactions and metabolic pathways involving lipids. Lipids were linked into a network employing the LIPID MAPS^®^ reaction explorer. To build this network, we used all measured lipids marked with grey dotted circles, while the statistically significant lipids were annotated with red dotted circles. All other lipids were kept to maintain the connectivity between other lipid classes.

To illustrate the connectivity of measured lipids, we performed pathway analysis. Because lipids, especially complex, are poorly represented in “classic” pathways, we decided to use LIPID MAPS^®^ reaction explorer to connect detected lipids into a network. Lipids were represented as a class instead of individual species. Figure 5 shows the results of this analysis. To build this network, we used all measured lipids marked with grey dotted circles, while the statistically significant lipids were annotated with red dotted circles. All other lipids were kept to maintain the connectivity between other lipid classes.

### 3.3 Implications of found lipid profile differences between ADC and SCC subjects

In order to provide better differential diagnosis and treatment methods, the discovery of molecular patterns of different lung cancer subtypes is needed. Thus, analysis of tissue samples seems to be a key to explore metabolic changes occurring at the site of the action where tumour development and growth occur. On the other hand, plasma samples must be also included since they are minimally invasively collected and routinely used in diagnostics.

In our study, we found the elevated level of PC 16:0/18:2 in tissue samples in ADC compared to SCC patients. It is in line with our previous research where tissue levels of PCs were increased in ADC (compared to SCC) (Kowalczyk et al., 2021). Other researchers also studied differences in lipidome of tumour samples between NSCLC subtypes, and some of the findings are in line with our results. Fan et al. observed elevated levels of PC 18:1_20:4, Cer d18:1/26:0, and several PEs and diradylglycerols (DGs) in ADC compared to SCC (Fan et al., 2020). Direct comparison of these results with our data is impossible, because the method we applied did not allow detection of either Cer or DG. Zhang et al. obtained similar results as they found the increased intensity of PC 34:0 in ADC compared to SCC (Zhang et al., 2019). Although PC 18:1_20:4 and PC 34:0 have different compositions of fatty acids than PC 16:0/18:2 we detected, all these molecules belong to the same lipid class and behave similarly. In contrast, You et al. found a lower level of PC 38:2 in ADC than in SCC samples. They also noted different tendencies in lipidome alterations, depending on the lipid class: PCs (LPC 20:1 and PC 38:2), free fatty acids (FAs) (FA 22:1 and FA 24:1) and carnitines (CARs) (CAR 2:0 and CAR 3:0) exhibited higher levels, while PEs (LPE 16:0 and PE 34:3), SM 35:2, and CAR 18:1 showed lower levels in ADC than in SCC (You et al., 2020). Our dataset contains six FAs, but none of them was found significantly different. However, despite the lack of significance, we observed a decrease in the signals of unsaturated FAs (FA 16:1, FA 18:1, FA 18:2, FA 18:3) and a very slight increase in the signals of saturated FAs (FA 16:0 and FA 18:0). Marien et al. discovered that eight phospholipids, including PC, (PC 40:2, PE 42:2, PE 44:5, PI 36:3, PI 36:4, PS 40:8, PS 42:9, SM 36:2) discriminated ADC and SCC, although the direction of change was not mentioned; therefore, a more detailed comparison of this results with our findings is not possible (Marien et al., 2015).

To the best of our knowledge, this study is the first to show significant differences in the level of several plasma LPAs between ADC and SCC patients. Patients suffering from ADC exhibited significantly higher levels of LPA 18:1, LPA 18:2, and LPA 20:4 than those diagnosed with SCC. LPAs originate from LPCs by the action of extracellular autotaxin (ATX) (Xie and Meier, 2004). ATX was found in many body fluids, e.g., plasma or malignant effusions (Tokumura, 2002). So far, there have been seven LPA receptors discovered (Gardell et al., 2006; Lin et al., 2010). LPAs act through activating cell proliferation, differentiation and migration, playing an important role in wound healing (van Corven et al., 1989; Willier et al., 2013; Aiello and Casiraghi, 2021). To date, many findings have shown overexpression of ATX in different pathological conditions, especially in different types of cancer (Yang et al., 2002; Kehlen et al., 2004; Wu et al., 2010). Elevated expression of ATX results in increased LPAs levels which are associated with tumour severity. Among patients with hepatocellular cancer, those with metastasis were characterized with higher serum LPAs level in comparison to those with no metastasis (Mazzocca et al., 2011). What is more, higher serum LPAs level was associated with larger tumour size as well as with poorer survival (Mazzocca et al., 2011). As for studies on lung cancer, LPAs was found to be involved in tumour microenvironment fibrosis. LPA-stimulated fibroblasts produced larger amounts of collagen (type I and VI) and fibronectin (Gudmann et al., 2019). Also, ATX-LPA axis was pointed to play a significant role in inflammation and lung cancer through the increase of proinflammatory cytokines (Valdés-Rives and González-Arenas, 2017). In our study, we noted reduced levels of three different LPAs in the serum of patients with ADC, compared to SCC. Since SCC is considered as more severe and characterized with poorer prognosis (Cooke et al., 2010; Fukui et al., 2015; Wang et al., 2020), our results suggest one of possible explanations for that and indicate the need for further investigation to better understand this phenomenon.

It is worth to note that around 4%–9% of NSCLC tumours contain mixed adenomatous and squamous pathologies within a single lesion, named adenosquamous cell carcinoma (AD-SCC), being the most lethal form of NSCLC with the worst prognosis (Hou et al., 2017). This indicates a potential phenotypic transition between ADC and SCC components in this pathologically mixed lung cancer (Yao et al., 2018). Moreover, there is an evidence that oxidative stress triggers ADC-to-SCC transdifferentiation, thus resistance to therapy. Some studies pointed out the essential role of extracellular matrix remodelling and metabolic reprogramming during this phenotypic transition. As reported, lipoxygenase (LOX) inhibitors and reactive oxygen species (ROS) significantly accelerate this transition. Although more profound research is needed to explain the exact mechanistic principles of this process, already our own evidence suggests that LOX downregulation results in decreased collagen deposition and extracellular matrix remodelling, leading to the transdifferentiation (Li et al., 2015; Yao et al., 2018). These indicate that balanced redox status is critical to control tumour plasticity and therapeutic response in NSCLC (Arfin et al., 2021), pointing to the need to explore changes in the oxPCs either in the transdifferentiation process or in AD-SCC patients.

OxPCs differentiate NSCLC subtypes on the tissue and plasma level. However, stronger differences were observed on plasma level. Three LCh-oxPCs (PC 16:0/20:4; OOH, PC 18:0/20:4; OH and PC 16:0/18:2; OH) were elevated in plasma of ADC patients in comparison to the SCC subjects, while:. in case of tissue samples only one SCh-oxPCs (PC 16:0/4:0; CHO) was significantly reduced in ADC subjects. Smaller differences in case of tumour tissue samples might be related to tumour heterogeneity and presence of different cellular and non-cellular components.

These results are very important since the relevance of glycerophospholipids, and oxidised phospholipids in lung functioning were already reported. Karki et al. pointed out that circulating and cell membrane oxPCs exhibit protective and deleterious effects on lung endothelium (Karki and Birukov, 2020). LCh-oxPCs were indicated as those with protective functions, while SCh-oxPCs as inducing harmful effects. However, the majority of these reports refer rather to the local activity of tissue oxPCs.

In our other work (Godzien et al., 2024), we found an elevation of oxPCs in NSCLC patients in comparison to the control group. Moreover, it has been reported that oxidative stress is elevated in ADC patients in comparison to SCC subjects (Gęgotek et al., 2016; Zalewska-Ziob et al., 2019), what stays in line with the changes observed in this study. The escalation of oxidative stress and impairment of the antioxidative defence system is greater in ADC patients, which can explain higher plasma levels of oxPCs in these subjects. However, in tumour tissue, we observed a reduced level of PC 16:0/4:0; CHO in ADC subjects. This might be due to already mentioned heterogeneity of tumour tissue or the fact that different oxidation products might exhibit different behaviour under a given condition. This concerns early and end-oxidation products, as well as different oxPCs fractions. Moreover, oxPCs may exhibit different effects depending on the concentration (Karki and Birukov, 2020). This clearly illustrates, that despite already available evidence, there is a need to even further explore the role of oxPCs and other oxidation products in NSCLC patients.

It is essential to mention that oxPCs were found to act as ligands for VEGF receptors (Mohammad and Srivastava, 2012). VEGF promotes tumour angiogenesis through its potent mitogenic effect on vascular endothelial cells (ECs). This links oxPCs with angiogenesis: it was demonstrated that oxPCs, precisely oxPAPC, an LCh-oxPC, stimulate angiogenic reactions in endothelial cells (Bochkov et al., 2017). This is particularly important, because VEGF receptors are known molecular targets for NSCLC therapy (Qu et al., 2018). Moreover, it was reported that hypoxia upregulates the protein levels of VEGF-A in lung cancer cell lines; however, the role of VEGF-A is distinct in ADC and SCC. VEGF-A protein levels were found significantly associated with tumour size and lymph node metastasis, and negatively correlated with the overall survival of ADC subjects, but not SCC patients (Qin et al., 2020). This, together with the differences in the profile of oxPCs between ADC and SCC patients that we observe, suggests that oxPCs should be evaluated as potential prognostic markers to monitor the effectiveness of the treatment and stop or prevent tumour growth. Among all tested lipids, only LPAs and oxPCs were found significantly altered. The linkage between these two groups of phospholipids is inflammation (Figure 5). It was reported that LPAs induce cytokines and interleukin 8 (IL-8) production, promote nuclear factor kappa-light-chain-enhancer of activated B cells (NF-κB) transcription and lymphocyte infiltration. This promotes inflammation and further production of inflammatory cells, white blood cells and interleukins. Inflammatory cells produce a highly oxidative environment, leading to ROS generation. Lipids subjected to the high concentration of ROS undergo oxidation, causing the formation of oxidation products, including also oxPCs. The elevated level of oxPCs (and other peroxidation products) results in excessive ROS generation by ECs. Finally, this increases inflammatory response even more.

Our data show elevated levels of LPAs in SCC patients. For this cohort of patients previous study showed elevated levels of inflammation factor NF-κB (Gęgotek et al., 2016). On the other hand, we observed higher levels of oxPCs for ADC subjects, and higher lipid peroxidation was reported for these patients (Gęgotek et al., 2016). Oxidative stress and inflammation can lead to cancerogenesis; however exact mechanisms underlying this process are distinct. Our results, combined with previously reported evidence, suggest that inflammation might be more involved in SCC development, while oxidative stress underlies ADC development. However, this statement can be a preliminary hypothesis that requires further examination in a larger group of patients, performing quantitative measurements of discriminating lipids together with determination of inflammatory and oxidative stress markers.

**FIGURE 6:**
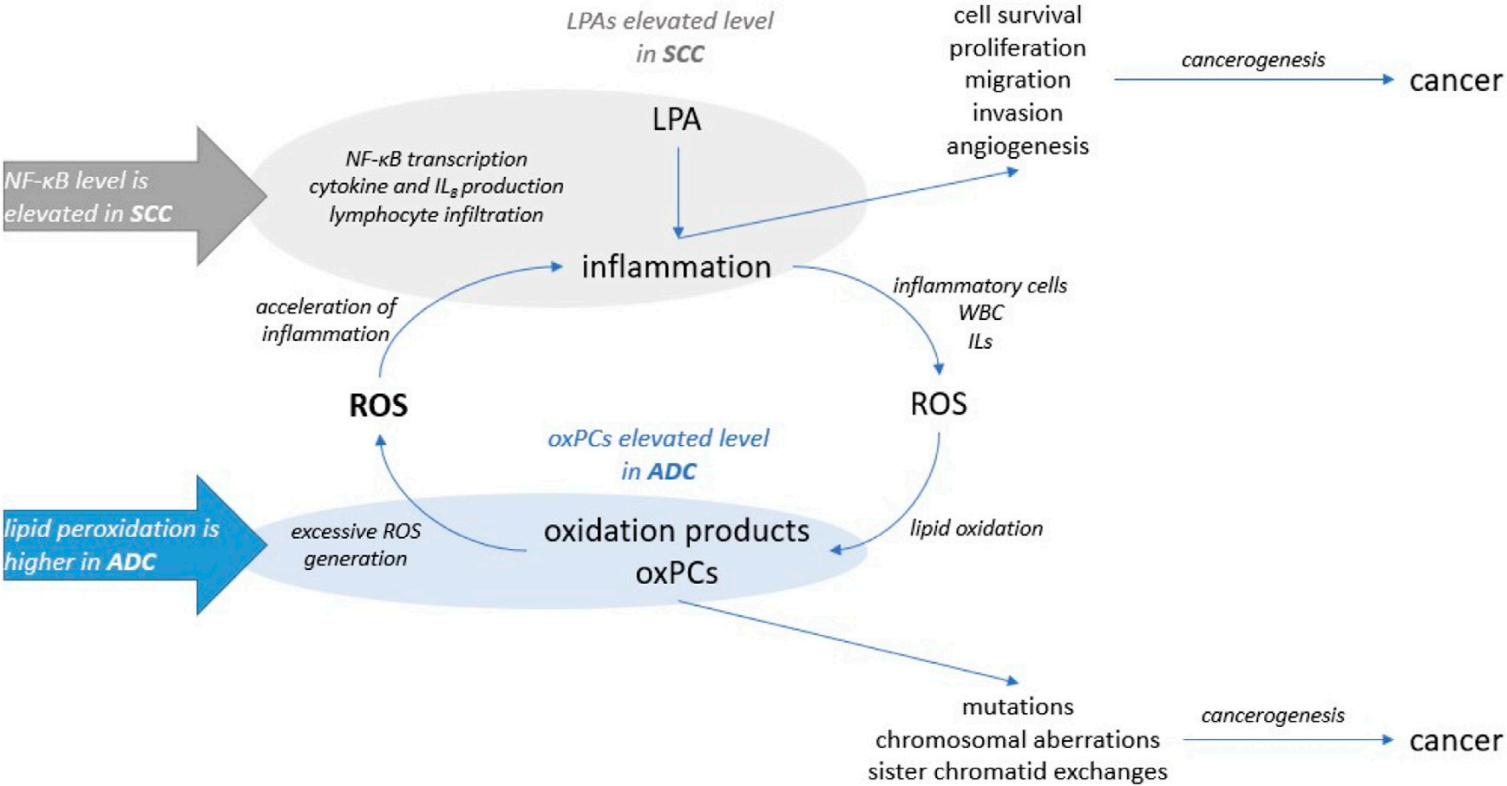
The involvement of LPA and oxPCs in the cancerogenesis: LPAs were linked to the inflammation and oxPCs were connected to the oxidative stress.

**FIGURE 7:**
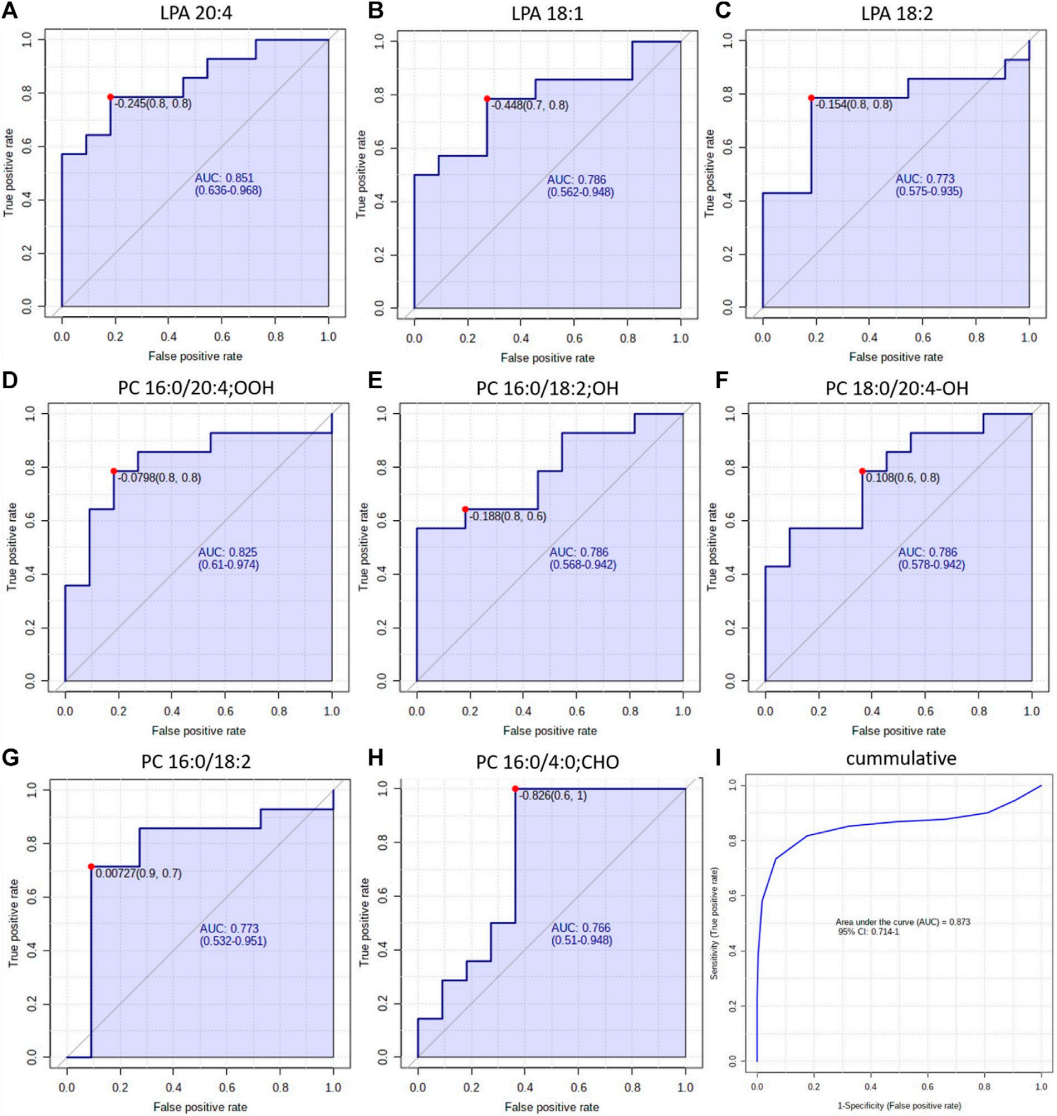
ROC curves obtained for each discriminating lipid in plasma (panels **(A–F)**) and tumour tissue panels **(G, H)**. Panel **(I)** shows the ROC obtained for all discriminating lipids simultaneously.

These results provide new insight into the mechanism underlying the development of both NSCLC subtypes. However, the ultimate goal of lipidomics study prognostic, diagnostic, predictive or therapeutic markers can be proposed (Carlomagno et al., 2017). As diagnosis of NSCLC subtypes poses a challenge, we decided to test discriminating LPAs and oxPCs as potential diagnostic markers. For this purpose, we constructed ROC curves for each discriminating lipid (Figure 7). Obtained AUCs were: 0.851, 0.786 and 0.773 for LPA 20:4, LPA 18:1 and LPA 18:2, respectively (Figure 7, panels A–C). For plasma oxPCs computed AUCs were: 0.825, 0.786 and 0.786 for PC 16:0/20:4; OOH, PC 16:0/18:0; OH and PC 18:0/20:4; OH respectively (Figure 6, panels D–F). For tumour PCs calculated AUCs were: 0.773 and 0.766 for PC 16:0/18:2 and PC 16:0/4:0; CHO, correspondingly (Figure 7, panels G and H).

Moreover, we tested different combinations of these lipids to create a biomarker model. The best results, with the highest AUC, were obtained for the combination of discriminating metabolites, i.e., AUC raised up to 0.873 (Figure 6, panel I). Other researchers used a similar strategy and tested the combined discrimination performance of several metabolites. For five DGs and one Cer, Fan et al. obtained AUC of 0.92 and 0.77 in the discovery and validation set, respectively (Fan et al., 2020). You et al. received even more robust results with AUC of 0.935 and 0.924 for discovery and validation cohorts, respectively. They used a relatively large set of metabolites, which included three carnitines, two free fatty acids, several phospholipids and five polar metabolites such as guanosine, creatinine and oxidised glutathione, among others (You et al., 2020). Zhang et al. performed ROC curve analysis over succinic anhydride, serine phosphorylcholine and PC 34:0, obtaining AUC of 0.827 (Zhang et al., 2019). Analysis of all these reported AUCs suggests that stronger values are obtained either for larger sample sets or for larger sets of discriminating metabolites. Therefore, the performance of our potential markers might be improved by validating them on a larger set of samples or by augmenting them with other metabolites, also polar. All these results show a clear potential of oxPCs and LPAs in the NSCLC subtypes diagnosis; however, they must be validated, covering proper quantification of these molecular lipid species on a larger cohort of patients. This should include a larger number of participants, but also patients with LCC, which were not included in this study due to low availability of the samples. Moreover, diagnostic utility of these glycerophospholipids must be tested in the context of presence of driver mutations such as, e.g., EGFRL858R, KRAS, ALK, among other.

## 4 Conclusion

Our results revealed differences in the profiles of oxPCs and LPAs between ADC and SCC patients. We observed elevated LPAs levels in SCC patients and increased levels of oxPCs in ADC subjects. These results align with publications reporting altered oxidative stress and inflammation markers in NSCLC subtypes. By combining our results with literature reports, we linked the observed increased level of LPAs with inflammation and noticed an increased level of oxPC with oxidative stress. All this suggests that inflammation might be more involved in the SCC development, while oxidative stress seems to underly the development of ADC. However, inflammation and oxidative stress are inherently connected; therefore, their impact on cancer development permeates each other. These results open a new line of research, pointing oxPCs and LPAs as potential markers and/or therapeutic targets in ADC and SCC.

## Figures and Tables

**Table:** 

**4-HNE**	4-hydroxynonenal
**ADC**	adenocarcinoma
**AD-SCC**	adenosquamous cell carcinoma
**ALK**	anaplastic lymphoma kinase
**ATX**	autotaxin
**BMI**	body mass index
**CAR**	carnitine
**Cer**	ceramide
**COPD**	chronic obstructive pulmonary disease
**DG**	diradylglycerol
**EC**	endothelial cell
**EGF**	epidermal growth factor
**EGFR**	epidermal growth factor receptor
**FA**	free fatty acid
**HILIC**	hydrophilic interactions chromatography
**HPLC**	high performance liquid chromatography
**IE-DDA**	iterative exclusion data-dependent analysis
**IGF-I**	insulin-like growth factor I
**IL-8**	interleukin 8
**LC**	liquid chromatography
**LCC**	large-cell carcinoma
**LCh-oxPC**	long-chain oxidised phosphocholine
**LC-MS**	liquid chromatography mass spectrometry
**LOX**	lipoxygenase
**LPA**	lysophosphatidic acid
**LPC**	lysophosphatidylcholine
**LPE**	lysophosphatidylethanolamine
**LPG**	lysophosphatidylglycerol
**LPI**	lysophosphatidylinositol
**MDA**	malondialdehyde
**MS**	mass spectrometry
**NF-κB**	nuclear factor kappa-light-chain-enhancer of activated B cells
**NSCLC**	non-small cell lung cancer
**oxPC**	oxidised phosphatidylcholine
**PC**	phosphatidylcholine
**PDFG**	platelet-derived growth factor
**PE**	glycerophosphoethanolamine
**PI**	glycerophosphoinositol
**PS**	glycerophosphoserine
**pTNM**	pathological tumour-node-metastasis staging
**QC**	quality control
**ROS**	reactive oxygen species
**RP**	reversed-phase chromatography
**RT**	retention time
**SCC**	squamous cell carcinoma
**SCh-oxPC**	short-chain oxidised phosphocholine
**SM**	sphingomyelin
**UHPLC**	ultra-high-performance liquid chromatography
**VEGF**	vascular endothelial growth factor

## Data Availability

The raw data supporting the conclusion of this article will be made available by the authors, without undue reservation.
