# Supplementary material for: First insight about the ability of specific glycerophospholipids to discriminate non-small cell lung cancer subtypes
Source: Front Mol Biosci. 2024 Apr 25;11:1379631. doi: 10.3389/fmolb.2024.1379631 (PMC11079276; doi:10.3389/fmolb.2024.1379631)
Supplement: Supplementary file 1 [file DataSheet2.PDF]

Matlab script used to perform Mann-Whitney U-test:

```
data: observations(patients)should be provided in individual columns, and
variables(metabolites)in lines. All data (regardless of sample grouping) must
be provided.
To provide information about sample grouping:
A: group 1 in the comparison
B: group 2 in the comparison
A=data (1:x) ;
B=data (x+1:y) ;
x – the number of the column of the last sample from group A
y- the number of the column of the last sample from group B
```

```
data=data';

A=data(1:8,:);
B=data(9:16,:);

[m,n]=size(data);
for i=1:n
pval(i)=ranksum(A(:,i), B(:,i));
end
p=pval';
```

Matlab script used to perform Benjamini-Hochber *p*-value correction:

```
p: Insert all raw p-values.
```

```
[n,m]=size(p);
pBH=zeros(1,n);
pcorregido=zeros(1,n);
[pord,orden]=sort(p);
pord=pord';
pcorregido(1,n)=pord(1,n);
for i=(n-1):-1:1;
    pcorregido(1,i)=(pord(1,i)/i)*n;
    if pcorregido(1,i)>pcorregido(1,i+1);
        pcorregido(1,i)=pcorregido(1,i+1);
    end
end
for i=1:n;
    pBH(1,i)=pcorregido(1,orden==i);
end
pBH=pBH';
```

R code used to perform Random Forest analysis and compute Gini score.

Packets needed to run the code:

```
library(readr)
library(dplyr)
library(tidyr)
library(stringr)
library(randomForest)
```

```
# Simple example on iris data -------------------------

data <- datasets::iris

set.seed(2000)

model <- randomForest(as.factor(Species) ~ ., data = data,
                      ntree = 2000, mtry = 2,
                      importance = TRUE)

results <- data.frame(Gini=sort(importance(model, type=2)[,], decreasing=T))

print(model$confusion)

print(results)

mean_decrease_acc <- data.frame(mean_decrease_acc = sort(importance(model, type=1)[,],
decreasing=T))

print(mean_decrease_acc)

oob_error_rate <- model$err.rate[nrow(model$err.rate), "OOB"]
```

```
print(oob_error_rate)


# Example on metabolomic data -----------------------


# Write the path to downloaded data below

path <- "./metabo_data_jg.csv"

path1 <- "C:/Users/bergi/OneDrive/Pulpit/CBK/materiały dla Krzysztofa/przykładowe dane/dane_dla_j.csv"


raw_data <- read_delim(path1)

str(raw_data)


metabo_data <- raw_data %>%

  mutate(group = gsub("_\\d+$", "", Compound))


# Adjusting column names

colnames(metabo_data) <- str_replace_all(colnames(metabo_data), "[[:punct:]]", " ")

colnames(metabo_data) <- gsub("-", "_", colnames(metabo_data))

colnames(metabo_data) <- gsub(":", "_", colnames(metabo_data))

colnames(metabo_data) <- gsub(" ", "_", colnames(metabo_data))

names(metabo_data) <- paste0('X', names(metabo_data))

metabo_data <- metabo_data[,-1]

str(metabo_data)



set.seed(2000)


metabo_model <- randomForest(as.factor(Xgroup) ~ ., data = metabo_data,

                             ntree = 2000,
```

```
                    #mtry = 2,
                    importance = TRUE)

metabo_model$confusion

metabo_results <- data.frame(Gini=sort(importance(metabo_model, type=2)[,], decreasing=T))
print(metabo_results)

metabo_mean_decrease_acc <- data.frame(mean_decrease_acc = sort(importance(metabo_model,
type=1)[,], decreasing=T))
print(metabo_mean_decrease_acc)

metabo_oob_error_rate <- metabo_model$err.rate[nrow(metabo_model$err.rate), "OOB"]
print(metabo_oob_error_rate)


# If your want to export csv file with the results, uncomment the lines below and write down desired
path

#export_path <- ""
#to_export <- data.frame(RowNames = rownames(metabo_results), metabo_results, row.names = NULL)
#write_csv(to_export, my_path)
```
